# Supplementary material for: Characterisation of 22445 patients attending UK emergency departments with suspected COVID-19 infection: Observational cohort study
Source: PLoS One. 2020 Nov 25;15(11):e0240206. doi: 10.1371/journal.pone.0240206 (PMC7688143; doi:10.1371/journal.pone.0240206)
Supplement: S1 Appendix — (PDF) [file pone.0240206.s001.pdf]

AFFIX PATIENT DETAILS LABEL HERE IF AVAILABLE

Name ☐ Male ☐ Female

Date of birth ☐☐:☐☐:☐☐☐☐

NHS Number ☐☐☐☐☐☐☐☐☐☐

Hospital Number ☐☐☐☐☐☐☐☐☐☐

DATE: ☐☐:☐☐:☐☐☐☐

TIME: ☐☐:☐☐

PRESENTING FEATURES:

☐ Shortness of breath ☐ Confusion ☐ Vomiting
☐ Cough ☐ Rash
☐ Fever ☐ Anosmia
☐ Sore throat ☐ Abdominal pain
☐ Headache ☐ Diarrhoea

REFERRAL SOURCE

☐ GP
☐ Self
☐ 111
☐ 999
☐ Other

PREVIOUS ☐ Flu Vaccine?[1] ☐ Oseltamivir?[2] ☐ Previous Attendance?[3]

ANTIBIOTIC THERAPY THIS ILLNESS? ☐ None
(Drug and duration)

SYMPTOM DURATION
(days)

CURRENT MEDICATION ☐ None

ALLERGIES TO MEDICATION ☐ None

MEDICAL HISTORY / CHRONIC DISEASE ☐ None

☐ Heart disease ☐ Diabetes ☐ Hypertension
☐ Renal impairment ☐ Active malignancy *(last 6 months)*
☐ Steroid therapy ☐ Immunosuppression
☐ Asthma ☐ Other chronic lung disease

RECENT TRAVEL HISTORY – last two weeks
(Country, duration and days since return)

LIFESTYLE ☐ Patient lives alone / no fixed abode ☐ Clinically obese ☐ Pregnant

☐ Tobacco user ☐ Vape user

☐ Known contact with Covid-19 case

Relationship to contact

WITHDRAWN CASE? Leaflet ☐ Verbal ☐

PAEDIATRIC ONLY

☐ Routine vaccinations? ☐ Taking feeds ☐ Parental anxiety? ☐ Premature[4]

PERFORMANCE STATUS (*tick one*)

☐ Unrestricted normal activity ☐ Limited strenuous activity, can do light ☐ Limited activity, can self care
☐ Limited self care ☐ Bed/chair bound, no self care

PANDEMIC RESPIRATORY INFECTION FORM

[1]Yes if any previous vaccine [2]Yes if any use of oseltamivir in current illness
[3]Yes if previous attendance at emergency dept. for this problem

[4]Premature defined as birth before 37 weeks gestation.

Version 7.0 27th Apr 2020

CLINICAL EXAMINATION

MOST LIKELY DIAGNOSIS? ☐ Influenza (Pandemic or seasonal) ☐ Covid-19 ☐ *Other (provide details)*

Respiratory Rate	
Pulse Rate	
Temperature	
Blood Pressure	
SaO_2	
Post exercise SaO2 *(if measured)*	
FiO_2	
Central capillary refill	Normal ☐ Abnormal ☐
GCS Total	☐ GCS individual scores not available
GCS-E	GCS-V GCS-M
A V	P U

☐ Severe respiratory distress[1] ☐ Respiratory exhaustion ☐ Severe dehydration

OTHER CLINICAL CONCERNS:

CXR	Not done ☐	Normal ☐	Abnormal ☐
ECG	Not done ☐	Normal ☐	Abnormal ☐

BLOODS TAKEN ☐

Na	K	Urea	Creat	Hb	Plate	WCC	Lymp	Neut	Lac - tate	CRP	D-dimer	Trop - onin

DISPOSITION AND CLINICAL PLAN

Oseltamivir ☐ *Antibiotic*

Antibiotic details

Clinician Name: Signature: Grade:

Disposed to: Date: Time:

PANDEMIC RESPIRATORY INFECTION FORM

[1]Severe respiratory distress (accessory muscles, tracheal tug, feeling of suffocation, apnoea)
