# Supplementary material for: Characterisation of 22445 patients attending UK emergency departments with suspected COVID-19 infection: Observational cohort study
Source: PLoS One. 2020 Nov 25;15(11):e0240206. doi: 10.1371/journal.pone.0240206 (PMC7688143; doi:10.1371/journal.pone.0240206)
Supplement: S2 Appendix — (PDF) [file pone.0240206.s002.pdf]

AFFIX PATIENT DETAILS LABEL HERE IF AVAILABLE

NHS Number ☐☐☐☐☐☐☐☐☐☐

Hospital Number ☐☐☐☐☐☐☐☐☐☐

DATE OF INITIAL VISIT: ☐☐:☐☐:☐☐☐☐

30 DAYS DUE AT: ☐☐:☐☐:☐☐☐☐

Mortality status

☐ Alive ☐ Dead → Date of death ☐☐ ☐☐ ☐☐☐☐ (d d m m y y y y)

Respiratory pathogen confirmed ☐ Influenza (Pandemic or seasonal)

☐ Covid-19 ☐ Other *(provide details)*

Date pathogen confirmed ☐☐ ☐☐ ☐☐☐☐ (d d m m y y y y)

Was patient admitted at initial assessment

☐ Yes ☐ No → ☐ Discharged home ☐ Transferred to other hospital ☐ Other / destination unknown

Admissions up to 30 days: *using patient's notes please complete as fully as possible, indicating dates for events and what support provided. Please include initial assessment if patient was admitted.*

Reason for admission	Ward	Admission dates	Respiratory support		Cardiovascular support		Renal support	
			Days	Type	Days	Type	Days	Type
	☐ Ward<br>☐ ITU<br>☐ HDU	Start ☐☐ ☐☐ ☐☐☐☐ (d d m m y y y y)<br>End ☐☐ ☐☐ ☐☐☐☐ (d d m m y y y y)						
	☐ Ward<br>☐ ITU<br>☐ HDU	Start ☐☐ ☐☐ ☐☐☐☐ (d d m m y y y y)<br>End ☐☐ ☐☐ ☐☐☐☐ (d d m m y y y y)						
	☐ Ward<br>☐ ITU<br>☐ HDU	Start ☐☐ ☐☐ ☐☐☐☐ (d d m m y y y y)<br>End ☐☐ ☐☐ ☐☐☐☐ (d d m m y y y y)						

30 day follow-up – for researcher use only

AFFIX PATIENT DETAILS LABEL HERE IF AVAILABLE

NHS Number ☐☐☐☐☐☐☐☐☐☐

Hospital Number ☐☐☐☐☐☐☐☐☐☐

Was a DNR decision made at any time between initial presentation and follow-up ☐ Yes ☐ No → Date ☐☐ ☐☐ ☐☐☐☐ (d d m m y y y y)

If patient experienced any events that did not require respiratory, cardiovascular or renal support, but that:

- **were life threatening,**
- **resulted in persistent or significant disability or incapacity**
- **prolonged hospitalisation**

Please add details below

Adverse events: using patient's *notes please complete as fully as possible, indicating dates for events and what support provided*

Event details	Event dates	Seriousness
	Start ☐☐ ☐☐ ☐☐☐☐ (d d m m y y y y)<br>End ☐☐ ☐☐ ☐☐☐☐ (d d m m y y y y) OR ☐ Ongoing	☐ Life threatening<br>☐ Persistent or significant disability or incapacity<br>☐ Prolongs hospitalisation
	Start ☐☐ ☐☐ ☐☐☐☐ (d d m m y y y y)<br>End ☐☐ ☐☐ ☐☐☐☐ (d d m m y y y y) OR ☐ Ongoing	☐ Life threatening<br>☐ Persistent or significant disability or incapacity<br>☐ Prolongs hospitalisation
	Start ☐☐ ☐☐ ☐☐☐☐ (d d m m y y y y)<br>End ☐☐ ☐☐ ☐☐☐☐ (d d m m y y y y) OR ☐ Ongoing	☐ Life threatening<br>☐ Persistent or significant disability or incapacity<br>☐ Prolongs hospitalisation

30 day follow-up – for researcher use only
