# Supplementary material for: Characterisation of 22445 patients attending UK emergency departments with suspected COVID-19 infection: Observational cohort study
Source: PLoS One. 2020 Nov 25;15(11):e0240206. doi: 10.1371/journal.pone.0240206 (PMC7688143; doi:10.1371/journal.pone.0240206)
Supplement: S5 Appendix — (DOCX) [file pone.0240206.s005.docx]

**S5 Appendix: Supporting Research Staff**

Marie Hyslop

Dan Beever

Samuel Keating

Kerry Wilson

Heather Dakin

Edwin Burkinshaw

Kirsty Pemberton

Tim Chater

Chris Turtle

Emily Turton

Matthew Bursnall

Mike Bradburn

Jennifer Petrie

Lizzie Swaby

Gemma Hackney

Judith Cohen
